# Dopaminergic Dysfunction Is More Symmetric in Dementia with Lewy Bodies Compared to Parkinson’s Disease

**DOI:** 10.3233/JPD-230001

**Published:** 2023-06-13

**Authors:** Tatyana Dmitrievna Fedorova, Karoline Knudsen, Jacob Horsager, Allan K. Hansen, Niels Okkels, Hanne Gottrup, Kim Vang, Per Borghammer

**Affiliations:** a Department of Nuclear Medicine and PET Centre, Aarhus University Hospital, Aarhus, Denmark; b Institute of Clinical Medicine, Aarhus University, Aarhus, Denmark; c Department of Nuclear Medicine and PET Centre, Aalborg University Hospital, Aalborg, Denmark; d Department of Neurology, Aarhus University Hospital, Aarhus, Denmark

**Keywords:** Parkinson’s disease, Lewy body disease, lewy body dementia, positron emission tomography computed tomography, dopaminergic imaging

## Abstract

**Background:**

The *α*-syn Origin site and Connectome model (SOC) proposes that *α*-synucleinopathies can be divided into two categories: the asymmetrical *brain-first,* and more symmetrical *body-first* Lewy body disease. We have hypothesized that most patients with dementia with Lewy bodies (DLB) belong to the *body-first* subtype, whereas patients with Parkinson’s disease (PD) more often belong to the *brain-first* subtype.

**Objective:**

To compare asymmetry of striatal dopaminergic dysfunction in DLB and PD patients using [^18^F]-FE-PE2I positron emission tomography (PET).

**Methods:**

We analyzed [^18^F]-FE-PE2I PET data from 29 DLB patients and 76 PD patients who were identified retrospectively during a 5-year period at Dept. of Neurology, Aarhus University Hospital. Additionally, imaging data from 34 healthy controls was used for age-correction and visual comparison.

**Results:**

PD patients showed significantly more asymmetry in specific binding ratios between the most and least affected putamen (*p* < 0.0001) and caudate (*p* = 0.003) compared to DLB patients. PD patients also had more severe degeneration in the putamen compared to the caudate in comparison to DLB patients (*p* < 0.0001) who had a more universal pattern of striatal degeneration.

**Conclusion:**

Patients with DLB show significantly more symmetric striatal degeneration on average compared to PD patients. These results support the hypothesis that DLB patients may be more likely to conform to the body-first subtype characterized by a symmetrical spread of pathology, whereas PD patients may be more likely to conform to the brain-first subtype with more lateralized initial propagation of pathology.

## INTRODUCTION

Parkinson’s disease (PD) and dementia with Lewy bodies (DLB) are *α*-synucleinopathies characterized by pathological accumulation and prion-like spreading of misfolded *α*-synuclein (*α*-syn), a pre-synaptic protein located throughout the brain [1, 2]. The origin and subsequent spreading of misfolded *α*-syn in these disorders has been debated and we recently proposed the *α*-Syn Origin site and Connectome model (SOC) to describe development of pathophysiology in *α*-synucleinopathies. SOC focuses on the *α*-syn pathobiology being the primary feature of pathogenesis in PD and DLB with special emphasis on the anatomical location of the first misfolded *α*-syn as well as the neural connectome that defines further propagation of the first pathological seeds [3].

The SOC model is partly based on the assumption that Lewy Body disorders are divisible into a body-first subtype and a brain-first subtype according to the initial origin site of *α*-syn pathology. In body-first patients, the *α*-syn pathology originates in the enteric nervous system with subsequent propagation to the CNS via the sympathetic and parasympathetic connectome, including projections from the dorsal motor nucleus of the vagus (DMV). Due to left-right autonomic innervation, retrograde propagation of seeds leads to bilateral pathology in the DMV and sympathetic structures. Ascending intracerebral propagation of pathology then leads to a relatively symmetric involvement of the substantia nigra (SN). In brain-first patients, pathological *α*-syn most often originates in one olfactory bulb or amygdala. In this case the propagation of pathology should be initially more asymmetric, since only one hemisphere is involved from the onset.

Rapid eye movement sleep behavior disorder (RBD) is a clinical feature that may be used to distinguish between brain-first and body-first patients. Most patients with RBD develop PD, DLB, or rarely multiple system atrophy (MSA) over time with RBD being widely recognized as a prodromal phase of these disorders [4]. Recent imaging studies provided evidence that RBD patients display severe peripheral autonomic dysfunction at the level of manifest PD patients [5, 6]. Furthermore, recently diagnosed PD patients that are categorized according to their RBD status into body-first (+RBD) vs. brain-first (-RBD) subtypes display correspondingly different profiles on a multimodal imaging battery assessing peripheral autonomic integrity [5, 7]. RBD patients convert to approximately equal numbers of PD and DLB patients [8]. However, most DLB patients suffer from RBD with polysomnography-confirmed prevalence ranging from 50–90% across studies [9, 10]. PD patients on the other hand have less frequent RBD with a prevalence of around 30–40% increasing with longer disease duration [11]. These observations led to the hypothesis that the majority of DLB patients belong to the body-first subtype, whereas 60–70% of PD patients belong to the brain-first subtype. Hence, according to the SOC model, the dopaminergic loss in de novo DLB patients should be more symmetric on average, when compared to de novo PD patients.

In the present study, we tested this hypothesis by analyzing striatal asymmetry in de novo DLB and PD patients, who underwent dopamine transporter [^18^F]-FE-PE2I positron emission tomography (PET).

## MATERIALS AND METHODS

### Study population

This retrospective study assessed all patients diagnosed with either DLB or PD at the Department of Neurology, Aarhus University Hospital during a 5-year period between February 1, 2017 and February 1, 2022. DLB patients were diagnosed by a neurologist with sub-specialization in dementia who determined whether sufficient clinical impairment was present to warrant a dementia diagnosis. Subsequently, the Fourth consensus report of the DLB Consortium criteria was used to establish the diagnosis of DLB [12]. We only included patients, who had had an [^18^F]-FE-PE2I scan as part of their diagnostic work-up. A total of 29 patients with DLB and 76 patients with PD had an [^18^F]-FE-PE2I scan and they constitute the primary patient cohort of this study. Furthermore, in-house [^18^F]-FE-PE2I data from 34 healthy controls was used for statistical comparison. The controls were recruited using newspaper advertisements from the general population with an emphasis on matching ages of controls to those of newly diagnosed PD patients (age 50–85). The institutional review board at Aarhus University Hospital granted access to patient files. Individual patient’s consent was waived by the institutional review board in accordance with Danish law due to the retrospective nature of thestudy.

### Information from patient files

Patient files were thoroughly reviewed, and the following information was extracted for all individuals: Age, sex, first contact to the hospital regarding DLB or PD suspicion, date of [^18^F]-FE-PE2I PET, and self-reported presence of motor and non-motor symptoms (postural instability, orthostatic hypotension, urinary incontinence, constipation, hyposmia, and depression). DLB patient files were specifically assessed for core and supportive clinical features as well as indicative and supportive biomarkers according to the DLB consortium diagnostic criteria of probable or possible DLB [12]. PD patient files were specifically assessed for supportive criteria, exclusion criteria and red flags according to the Movement Disorder Society (MDS) Clinical Diagnostic Criteria for PD [13]. All [^18^F]-FE-PE2I scans were assessed by an expert reviewer and patients with normal scans were excluded. Healthy controls had no history of neurological disease and a normal neurological examination. Exclusion criteria for controls were cardiac disorders, kidney or liver failure, psychiatric disorders, use of psychiatric or illicit drugs, and alcoholism. Cognitive function was assessed in all controls and MoCA scores < 26 led to exclusion from the study.

### [^18^F]-FE-PE2I PET/CT acquisition

The radiosynthesis of [^18^F]-FE-PE2I was described previously [14]. No patients or controls took medications containing amphetamine, modafinil, or other dopamine transporter ligands prior to the [^18^F]-FE-PE2I PET/CT. The scan procedure was identical for both patients and controls. A 10-min PET data acquisition (list mode) on a Siemens Biograph Vision 600 PET/CT scanner (Siemens Healthcare, Erlangen, Germany) was initiated precisely 30 min after i.v. injection of 200 MBq [^18^F]-FE-PE2I, immediately preceded by a low-dose CT scan for attenuation correction and anatomical standardization. Images were reconstructed using an iterative OSEM algorithm with resolution recovery (TrueX), 8 iterations, 5 subsets, and all-pass filter.

### Image analysis

All [^18^F]PE2I PET/CT scans were interrogated qualitatively and semi-quantitatively by a neuro-specialized nuclear medicine physician with extensive expertise in reading dopamine scans (PB). In short, a visual, qualitative assessment of each scan was performed, and “neuro-degenerative” patterns were noted, such as asymmetric loss and/or predominant posterior putaminal loss of striatal binding. A co-registered CT was used to rule out structural causes of reduced striatal binding, such as lacunar strokes and dilated perivascular spaces. A semi-quantitative analyses was then conducted, as described by Marner and colleagues [15]. In short, an automated segmentation algorithm based on a previously created and published atlas was used to delineate volumes of interest (VOI) on static [^18^F]-FE-PE2I PET/CT images [16]. VOIs were defined for the left and right caudate nucleus and putamen as well as the cerebellum grey matter, which was used as reference region. CT images were transformed to an MNI template, and the VOI segmentations were obtained after matching of the PET image to the CT image and applying the same transformation to template space. Averaged count values from the VOIs were extracted after reverse transformation of the PET image to native space. Specific binding ratios (SBR) for left and right putamen and caudate nuclei were calculated as VOI/(reference region –1). Furthermore, putamen/caudate ratios (put/cau) were calculated separately for each hemisphere. The asymmetry index (AI) was calculated as follows: (least affected putamen – most affected putamen)/(least affected putamen + most affected putamen), (least affected caudate – most affected caudate)/(least affected caudate + most affected caudate). Since dopamine transporter density is known to decline with age, we adjusted SBR values for both patient groups using estimates of the age-dependent decline from the healthy controls. Linear regression analyses were performed on putamen and caudate SBR values separately for each hemisphere in healthy controls. Following that, putamen and caudate z-scores was then calculated by comparing patients SBRs to an in-house dataset of 34 healthy, aged controls, who had [^18^F]PE2I PET using the same PET camera and identical analyses methodology. A scan was deemed to be pathological, if both the qualitative assessment and semi-quantitative values showed unequivocal abnormalities compatible with a neurodegenerative Lewy body disorder in the absence of competing causes of decreased PE2I binding such as lacunar infarcts.

### Statistical analysis

Statistics were performed with Prism version 9 (GraphPad Software, La Jolla, USA). Normality was assessed using D’Agostino & Pearson normality test, Shapiro-Wilks test, Kolmogorov-Smirnov test, and Q-Q plots. Student’s unpaired *t*-test was used to compare numerical and normally distributed variables. In case of non-normal distribution, we used Mann-Whitney test. F tests were used to compare variances between groups. In case of differing variances, we used Welch’s correction. Kruskal-Wallis test was used to compare age distribution between groups. Chi-squared test was used to compare sex distribution between groups as well as prevalence of non-motor symptoms. Pearson correlation coefficient was calculated to assess the relationship between age and [^18^F]-FE-PE2I PET/CT in putamen and caudate. Linear regression analyses were used to adjust for age differences between groups. Mann-Whitney test was performed to assess differences in asymmetry between the sexes. Normally distributed data is presented as mean±SD, non-normally distributed data as median (range). Prevalence of non-motor symptoms are presented as a percentage. Statistical significance was defined as *p* < 0.05, and no multiple comparison corrections were performed.

## RESULTS

**Fig. 1:**
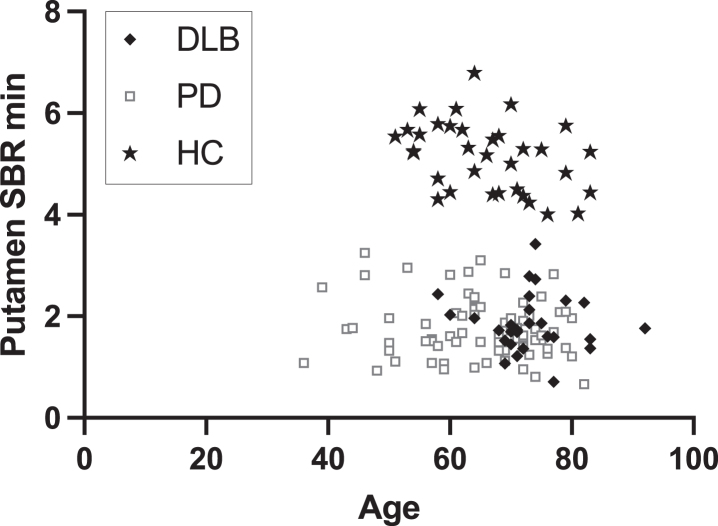
Distribution of most affected putamen [^18^F]-FE-PE2I specific binding ratio (SBR) and age in DLB patients, PD patients, and controls.

**Fig. 2:**
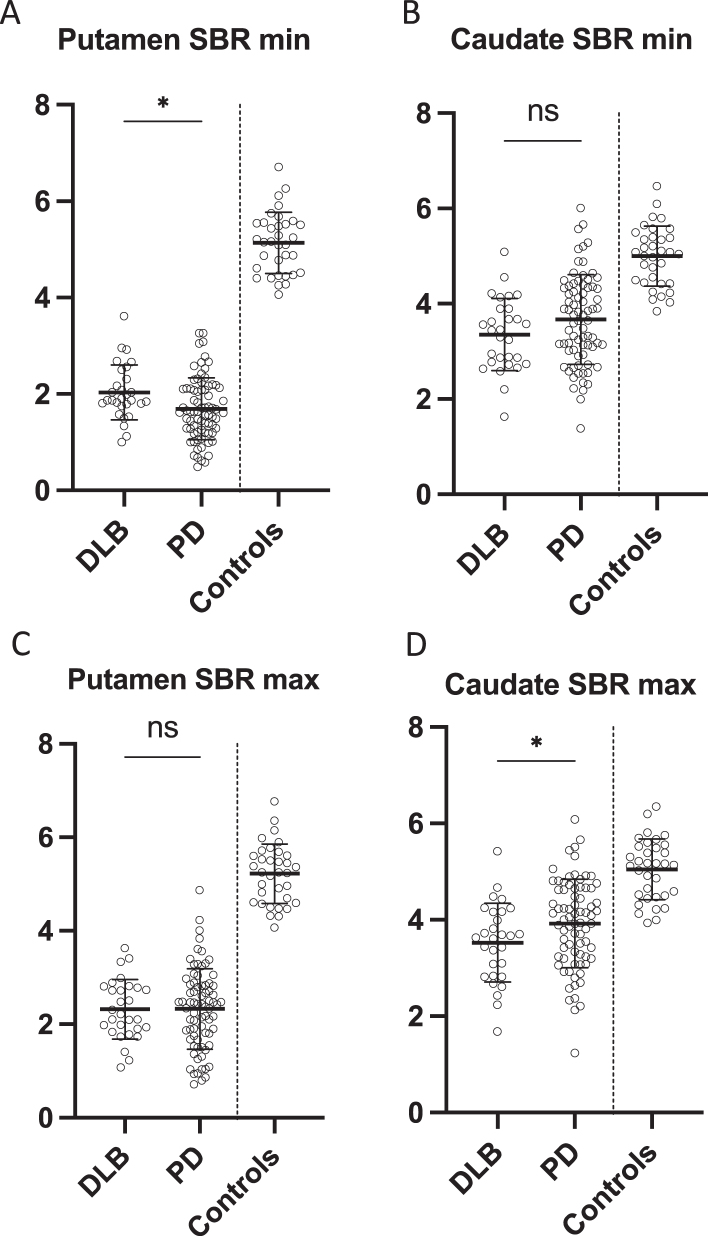
Most affected specific binding ratio in A) putamen and B) caudate in DLB patients, PD patients, and controls. Least affected specific binding ratio in D) putamen and E) caudate in DLB patients, PD patients, and controls. Data is presented as mean±SD. *p < 0.05. ns, non-significant; SBR, specific binding ratio.

**Fig. 3:**
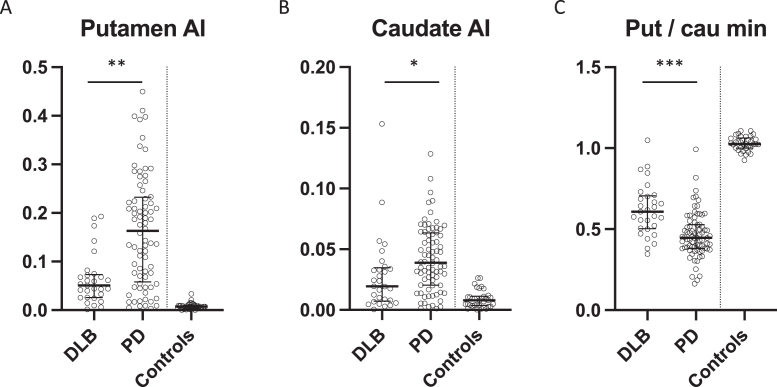
Asymmetry indexes from A) putamen and B) caudate in DLB patients, PD patients, and controls, presented as median±IQR. C) Most affected putamen to most affected caudate specific binding ratio, presented as median±IQR. *p = 0.0035. **p = 0.00002, ***p = 0.000004. AI, asymmetry index; Put, putamen; Cau, caudate.

**Fig. 4:**
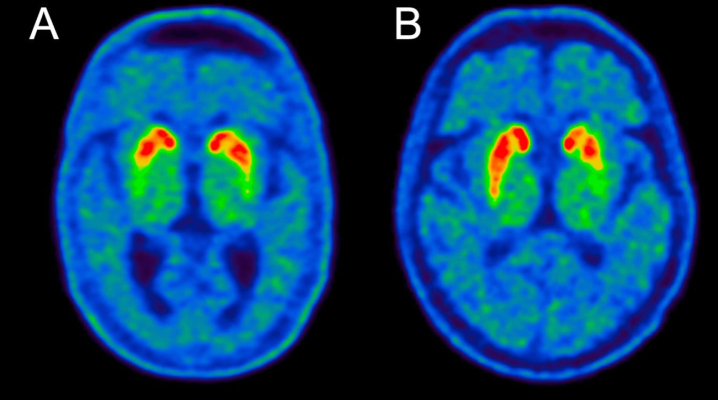
-FE-PE2I PET image examples from A) a DLB patient and B) a PD patient. Note the symmetric loss in the DLB patient. The putamen AI values were 0.05 for the DLB patient and 0.17 for the PD patient, which are representative of the medians of the two groups (see Fig. 3A).

We included 29 DLB patients and 76 PD patients. Demographic data and symptoms are presented in Table 1. Imaging data is presented in Table 2 and Figs. 1–4. Of the 29 DLB patients 28 had a diagnosis of probable DLB, and one patient had possible DLB, due to only having one indicative biomarker and no core clinical features. Of the 76 PD patients, 65 had a diagnosis of clinically established PD whereas 11 patients had clinically probable PD.

**Table 1:** Demographic and clinical characteristics of DLB, PD patients, and controls

	Controls (*n* = 34)	DLB patients (*n* = 29)	PD patients (*n* = 76)	p
Age (y)	67 (51–83)	73 (58–92)	68 (36–83)	**0.001**
Sex (male/female)	15/20	23/6	47/29	0.02
Timing of scan (months)^*^	N/A	4 (0–36)	3 (0–139)	0.41
Symptoms				
Constipation (y/n; %)	N/A	15/14; 52	27/49; 36	0.13
Urinary incontinence (y/n; %)	N/A	6/23; 21	13/63; 17	0.67
Postural instability (y/n; %)	N/A	13/16; 45	12/64; 16	**0.0018**
Depression (y/n; %)	N/A	10/19; 34	13/63; 17	**0.05**

^*^Time between first contact to the hospital regarding DLB/PD complaints and [^18^F]-FE-PE2I PET/CT scan. Results are presented as median (range) or number of individuals in each category; percentage of individuals who reported presence of a specific symptom.

**Table 2:** [^18^F]-FE-PE2I PET/CT results from DLB patients, PD patients, and controls. All reported results here are age-corrected

	DLB (*n* = 29)	PD (*n* = 76)	p^*^	Controls (*n* = 34)
Most affected putamen SBR	2.03±0.57	1.69±0.64	**0.01**	5.14±0.63
Least affected putamen SBR	2.32±0.64	2.33±0.86	0.97	5.22±0.64
Most affected caudate SBR	3.35±0.76	3.67±0.94	0.11	5.00±0.63
Least affected caudate SBR	3.53±0.82	3.92±0.92	**0.04**	5.05±0.63
Most affected put/cau ratio	0.61 (0.35–0.70)	0.45 (0.16–0.83)	**<0.0001**	1.03 (0.93–1.11)
Least affected put/cau ratio	0.67±0.17	0.59±0.16	**0.02**	1.04±0.04
Putamen AI	0.05 (0–0.19)	0.16 (0–0.45)	**<0.0001**	0.01 (0–0.03)
Caudate AI	0.02 (0–0.15)	0.04 (0–0.13)	**0.004**	0.01 (0–0.03)

*p-value signifies the comparison of DLB and PD patients. Results are presented as mean±SD or median (range). AI, Asymmetry index; SBR, Specific binding ratio; Put, putamen; Cau, caudate.

DLB patients were significantly older than both controls and PD patients with a median difference of five years compared to PD patients, whereas PD patients and controls did not differ in age. One DLB patient was older than the oldest control whereas nine PD patients were younger than the youngest healthy control (Fig. 1). The sex distribution was skewed with a higher proportion of women in healthy controls and a higher proportion of males in the DLB group. Time between the first visit to the hospital regarding neurological symptoms and [^18^F]-FE-PE2I PET/CT scan was not different between the groups. When assessing the amount of reported non-motor symptoms DLB patients had significantly more frequent postural instability and depression, and a trend towards more frequent constipation.

PD patients had significantly lower SBR in the most affected putamen compared to DLB patients (*p* = 0.005) whereas DLB patients had significantly lower SBR in the least affected caudate compared to PD patients (*p* = 0.04) (Fig. 2A+D). There were no differences in the least affected putamen and most affected caudate SBRs (Fig. 2B, C). The put/cau ratio was significantly different between the patient groups (*p* < 0.0001 for the most affected side and *p* = 0.01 for the least affected side) with the putamen being much more severely affected than the caudate in PD patients compared to DLB patients (Fig. 3C).

Furthermore, PD patients had significantly more asymmetrical reductions in nigrostriatal innervation compared to DLB patients (Figs. 3 and 4). This finding was most striking in the putamen where PD patients had a two-fold increase in the AI compared to DLB patients (*p* < 0.0001) (Fig. 3A). The difference in caudate AI also showed a two-fold increase in the PD group but the level of significance was smaller due to larger data variance (*p* < 0.01) (Fig. 3B).

*Post-hoc* analysis of differences in AI according to sex showed no differences in male vs. female in both DLB and PD groups (*p* = 0.93). The healthy controls showed an age-related decline in [^18^F]PE2I binding per year of 0.6% (right putamen), 0.5% (left putamen), 0.8% (right caudate), 0.7% (left caudate) (Supplementary Figure 1). The linear regression lines shown in the figure were used to adjust for the age difference between the two patient groups.

## DISCUSSION

We used dopamine transporter imaging, [^18^F]-FE-PE2I PET, to show that patients with DLB have significantly more symmetric striatal degeneration compared to PD patients. Furthermore, the putamen/caudate ratio of degeneration was markedly increased in PD patients suggesting that DLB patients have a more evenly distributed pathology throughout the striatum, whereas the degeneration in PD patients preferentially affects the putamen.

It has been shown previously that a pathological putamen AI in PD at baseline becomes increasingly less pathological with disease progression, since the least affected striatal regions “catch up” [17]. Thus, more severe degeneration could theoretically have caused the DLB group to have more symmetric dopaminergic dysfunction on the images. However, this was not the case in our data. Instead, the PD group showed the most pathological putamen SBR in the most affected hemisphere, suggesting that the dopaminergic degeneration in the PD patients was more advanced than in the DLB patients (Fig. 2A). Also, the time from the patient’s first healthcare contact to the [^18^F]-FE-PE2I PET scan was equal between the groups. These findings suggest that the “dopaminergic disease stage” was equal in PD patients and DLB patients.

Dopamine transporter density is inversely correlated with age in healthy individuals with older subjects having a lower signal on [^123^I]FP-CIT SPET imaging [18] and we found the same correlation in [^18^F]-FE-PE2I PET data. DLB patients were on average five years older than PD patients, and this could lead to an overestimation of their disease severity. We performed an age-adjusted estimation of the [^18^F]-FE-PE2I PET signal in both patient groups to avoid this issue. When comparing the putamen and caudate dopamine transporter PET signal without adjusting for age, the putamen signal was not different between groups, but the caudate signal on both sides was decreased in DLB patients compared to PD patients (data not shown).

Our findings align with the predictions of the SOC model, i.e., that a higher proportion of DLB patients have body-first etiology, which results in more symmetrical dopaminergic degeneration. In contrast, about two-thirds of PD patients are predicted to have brain-first Lewy body disease, and such patients are predicted to have generally more asymmetrical degeneration.

Our findings support similar results from previous studies. One study reported more symmetrical motor symptoms in DLB patients compared to PD patients [19]. A smaller imaging study compared striatal dopamine transporter density in PD patients and DLB patients using [^123^I]FP-CIT SPECT imaging [20]. They reported increased asymmetry in the putamen of PD patients in accordance with our findings. Furthermore, they also reported decreased putamen/caudate ratios in the PD group compared to DLB patients as shown in our data. Another study also found that the putamen was more severely degenerated than the caudate in PD patients compared to DLB patients [21].

Interestingly, a recent study investigated conversion rates for patients with isolated RBD and found that patients with a higher asymmetry on dopaminergic imaging of the caudate nucleus had a higher risk of conversion to DLB whereas more symmetrical caudate imaging led to conversion to PD [22]. This seems to contradict the findings here, thus more studies are needed to resolve these discrepant findings. Furthermore, a study of dopamine tracer imaging in recently diagnosed PD patients with and without RBD examined two distinct cohorts. They found a consistent difference on dopaminergic PET and SPECT imaging between patients suffering from RBD who were thought to belong to the body-first subtype and patients without RBD who were thought to belong to the brain-first subtype. Patients with RBD had more symmetrical striatal degeneration compared to patients without RBD [23].

Some limitations need to be addressed. The DLB and PD patients were not assessed for presence of RBD at time of diagnosis, so we were unable to confidently divide them into brain/body first categories. Thus, our patient groups most likely comprise mixed brain-first and body-first patients. Another limitation was the lack of detailed clinical information on participants. Unfortunately, we only had access to the electronic patient files and were not able to qualify disease severity in further detail, or evaluate cognitive, motor, and non-motor symptoms in a standardized research setting. Among other things, it would have been interesting to investigate whether the level of cognitive dysfunction in DLB patients correlated with caudate dopaminergic degeneration as has been proposed previously [21]. With regards to non-motor symptoms, we would have expected to see a more severe non-motor phenotype in the DLB group as seen in previous studies indicating that body-first PD patients have a more severe peripheral dysfunction [7, 24]. We did see more postural instability and depression in DLB patients and a trend towards more constipation, but no differences were found in other non-motor symptoms. However, all symptom assessments were subjective based on patient complaints and a more detailed objective evaluation of these symptoms could have been preferrable. Lastly, the DLB group was 5 years older on average compared to the PD group, which reflects a prospective recruitment of eligible subjects during a set time period since PD patients are diagnosed 5–10 years earlier than patients with DLB [25]. To correct for age-related decline in striatal PE2I binding, we applied a linear correction, which is the most commonly used method. It is however possible that the age-related decline follows a non-linear trajectory. Since the main PE2I parameter in this study was asymmetry index, it seems unlikely that a difference between linear and non-linear age correction would affect our overall results, since the asymmetry index itself is not affected by variable age-correction methods.

In conclusion, we confirmed that DLB patients exhibit more symmetric striatal degeneration on dopamine transporter PET around the time of diagnosis when compared to PD patients. These results could suggest that DLB patients are more likely to have a body-first etiology compared to PD patients, as predicted by the SOC model. These findings should be replicated in future studies, preferably with larger samples of well characterized PD and DLB patients with known RBD status and [^123^I]MIBG cardiac scintigraphies for better stratification into brain/body first subtypes. Additionally, longitudinal studies would shed light on how these differences in lateralization change with time.

## CONFLICT OF INTEREST

The authors have no conflict of interest to report.

## DATA AVAILABILITY

The data supporting the findings of this study are available on request from the corresponding author. The data are not publicly available due to privacy or ethical restrictions.

## Supplementary Material

Supplementary Material
