# Supplementary material for: Dopaminergic Dysfunction Is More Symmetric in Dementia with Lewy Bodies Compared to Parkinson’s Disease
Source: J Parkinsons Dis. 2023 Jun 13;13(4):515–23. doi: 10.3233/JPD-230001 (PMC10357144; doi:10.3233/JPD-230001)
Supplement: Supplementary Material [file jpd-13-jpd230001-s001.pdf]

Supplementary Material

Dopaminergic Dysfunction Is More Symmetric in Dementia with Lewy Bodies Compared to Parkinson's Disease

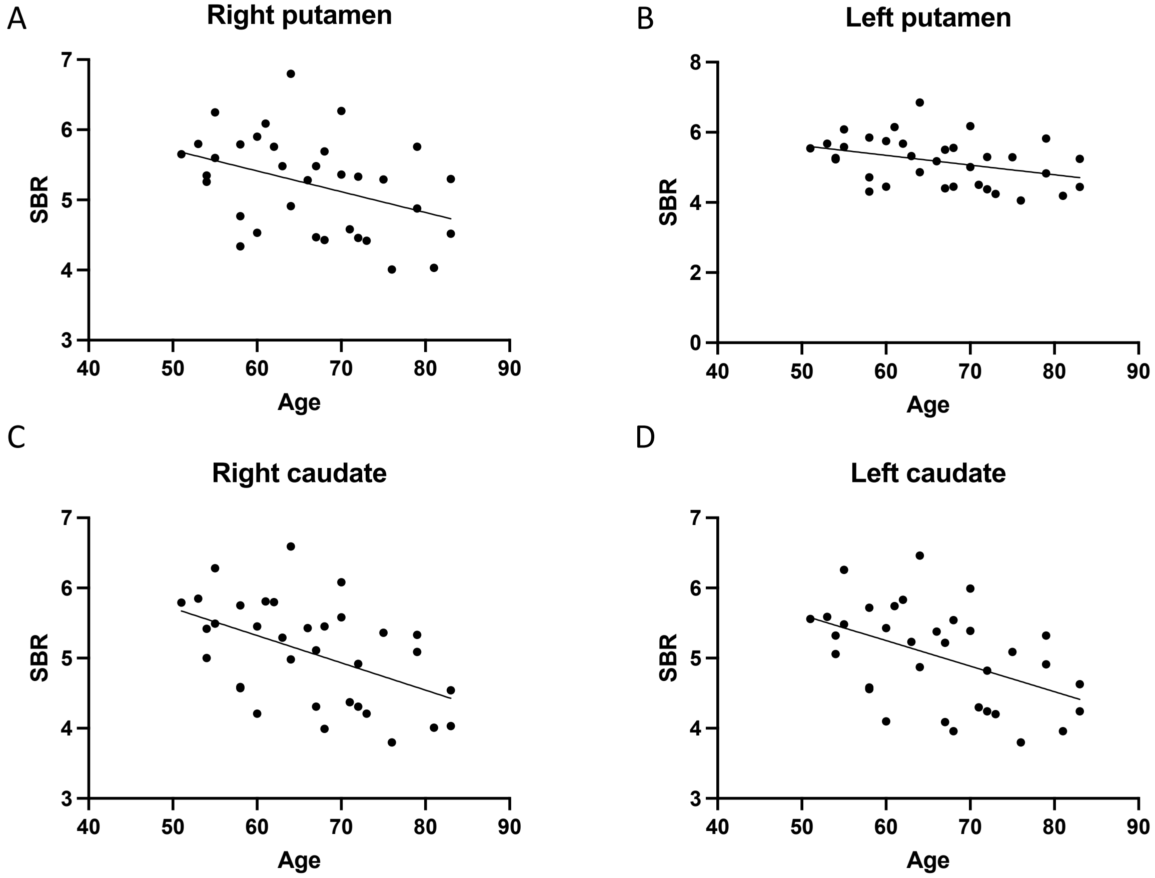

Supplementary Figure 1. Correlation between age and signal intensity in A) right putamen, B) left putamen, C) right caudate, and D) left caudate in healthy controls. All correlations were significant with p-values < 0.05. Linear regression equations were as follows: A) Y = -0,02963*X + 7,191; B) Y = -0,02770*X + 7,007; C) Y = -0,03892*X + 7,658; D) Y = -0,03640*X + 7,434. SBR, specific binding ratio.
